# Configuration of a Combined Microencapsulated Essential Oil Antimicrobials With Modified Atmosphere Biodegradable Films for Shelf‐Life Extension of Table Grapes

**DOI:** 10.1002/fsn3.71176

**Published:** 2025-11-21

**Authors:** Xueyan Yun, Lu Zhang, Tungalag Dong, Hongmei Zhang, Tao Sun, Jian Hu, Peifang Cheng

**Affiliations:** ^1^ College of Food Science and Engineering Inner Mongolia Agricultural University Hohhot People's Republic of China; ^2^ Engineering Research Center of Comprehensive Utilization of Livestock By‐Products Inner Mongolia Agricultural University Hohhot People's Republic of China; ^3^ China‐Mongolia Biomacromolecule Application “Belt and Road” Joint Laboratory Inner Mongolia Agricultural University Hohhot People's Republic of China

**Keywords:** microencapsulated essential oil, passive modified atmosphere packaging, preservation, table grape

## Abstract

Fresh grapes are susceptible to deterioration during storage due to their vigorous respiratory metabolism and the potential for microbial contamination. This study prepared three types of microcapsules containing essential oils derived from peppercorn and oregano, as well as their composite. Furthermore, we investigated the effects of these microcapsules combined with passive modified atmosphere packaging on the preservation quality and shelf life of table grapes stored at 12°C for 28 days. Five treatments were administered, including PCL/PLLA film without EO microcapsules and control groups (no packaging), PEOM + PCL/PLLA, OEOM + PCL/PLLA and CEOM + PCL/PLLA. The results indicated that the combination of CEOM with Polycaprolactone/Poly (L‐lactic acid) PCL/PLLA film (CEOM + PCL/PLLA) exhibited notable antibacterial and preservation properties. It effectively maintained optimal storage conditions of 6.4% oxygen and 14% carbon dioxide for the grapes over 28 days, significantly reduced the abundance of major spoilage bacteria, including *Penicillium* and *Rhizobium,* decreasing from 38% on the first day to 0%, and resulted in a total fungal count of only 2.3 × 10^3^ CFU·g^−1^, which is half that of the control group lacking EO microcapsules. The synergistic application of the CEOM and passive modified atmosphere packaging (PMAP) effectively extended the shelf life of table grapes from 12 to 28 days.

## Graphical abstract

This study illustrates the synergistic preservation effect of incorporating composite essential oil microcapsules (CEOM) into a passive modified atmosphere packaging (PMLAP) film for table grapes. The CEOM‐PMLAP system autonomously adjusts the internal atmosphere to an optimal equilibrium of 6.4% O_2_ and 14% CO_2_, effectively inhibiting the proliferation of predominant spoilage microorganisms such as *Penicillium* and *Rhizobium*. This suppression results in a 50% decrease in total fungal count compared to the control group. Consequently, this innovative combination significantly prolongs the shelf life of table grapes from 12 days to 28 days while preserving their high quality.

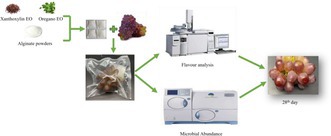

## 1 Introduction

Grapes ( *Vitis vinifera* L.) represent a significant global berry fruit, distinguished by their rich nutritional constituents, including vitamins, resveratrol, and anthocyanins. These compounds collectively contribute to the notable dietary and health benefits associated with grape consumption (Li, Bi, et al. 2024). However, due to their vigorous metabolic activity and susceptibility to microbial infections during storage, table grapes are particularly prone to decay, resulting in a deterioration of quality (Kai et al. 2024). In the context of grape storage, chemical preservatives such as sulfur dioxide are commonly utilized. Nevertheless, these sulfur‐containing compounds are highly prone to residual chlorine, thereby posing a threat to food safety. Consequently, the advancement of environmentally friendly biological control technologies has emerged as a primary focus in contemporary research on post‐harvest grape preservation.

Passive modified atmosphere packaging (PMAP) has been widely used for the preservation of vegetables and fruits due to its convenience and effective process (Tejedor‐Calvo et al. 2020). However, the prevalent use of non‐degradable synthetic polymers in PMAP raises considerable environmental concerns. Polycaprolactone (PCL), a biodegradable and biocompatible polyester, has gained interest as a sustainable alternative, yet its application is hindered by low mechanical strength, high gas permeability, and cost (Wang et al. 2025; Guo et al. 2025; Qin et al. 2024). It is well‐established that the incorporation of oxygen‐barrier material into polymer matrices can significantly modify the gas permeability of the resulting substance (Polevaya et al. 2020). Blending PCL with high‐barrier polymers presents a viable approach to tune material properties. Poly (L‐lactic acid) (PLLA), another biodegradable polymer, offers excellent mechanical strength and low gas permeability, making it a suitable candidate for enhancing PCL‐based films (Wang et al. 2023; Liu, Yang, et al. 2024). Although studies have demonstrated the effectiveness of such PMAP systems in maintaining desirable gas compositions and extending the shelf life of various fruits (Sun et al. 2024; Souza et al. 2022; Paulsen et al. 2019; Zeng et al. 2018). However, the antimicrobial efficacy of PMAP remains relatively limited, which may result in moisture accumulation during the packaging process and subsequently promote microbial growth. This limitation drives the need to integrate PMAP with antimicrobial strategies to achieve synergistic preservation effects.

Plant essential oils (EOs), such as peppercorn (PEO) and oregano (OEO) essential oils, are recognized as safe and effective natural antimicrobials, offering an alternative to synthetic preservatives (Zhi et al. 2021). Their efficacy stems from complex active compounds that inhibit microbial growth and exhibit antioxidant activity (Wei, Li, Qin, Wang and Zhong 2024; Luo et al. 2024). However, the high volatility and susceptibility of EOs to environmental factors limited their direct application in food preservation (Napiórkowska et al. 2023). Microencapsulation has been established as an effective strategy to overcome these challenges by protecting the active components (Zhu et al. 2024; Dima et al. 2016; Hasheminejad and Khodaiyan 2020), and sustaining their antimicrobial efficacy over time, as demonstrated by the long‐term bioactivity of OEO against common pathogens (Wanli et al. 2022).

The integration of microencapsulated essential oils (EOs) into PMAP is a promising strategy to enhance its antimicrobial efficacy. Nanocomposites often rely on inorganic nanoparticles for antimicrobial activity, which may face challenges including nanoparticle aggregation (Can et al. 2023), potential cytotoxicity concerns (Mohammad et al. 2020), and higher production costs due to complex synthesis processes (Karpov et al. 2022). In contrast, microencapsulated EOs utilize natural, food‐grade wall materials and allow for controlled release of active compounds (Infante et al. 2022; Huma et al. 2023). Since peppercorn and oregano EOs are effective against spoilage microbes, this study focuses on combining them with PMAP for grape preservation—an innovative approach that has not been thoroughly investigated.

This study aimed to develop microcapsules containing peppercorn and oregano essential oils and their composites, and to evaluate their effects of combining PMAP on the preservation quality and shelf life of table grapes stored at 12°C for 28 days.

## 2 Materials and Methods

### 2.1 Materials

PEO and OEO (purity > 99%) were obtained from Chenguang Biotech Group Co. Ltd. (Hebei, China). Sodium alginate, thiobarbituric acid, gallic acid and other reagents were acquired from Sinopharm Chemical Reagent Co Ltd. (Shanghai, China). All other chemical reagents were of analytical grade and were purchased from domestic reagent suppliers. PCL/PLLA films were self‐prepared in the functional packaging material laboratory (Thickness: 30 μm; Oxygen permeability: 6.60 × 10^−8^ cm^3^‧m‧m^−2^‧d^−1^‧Pa^−1^; Carbon dioxide permeability: 0.31 × 10^−6^ cm^3^‧m‧m^−2^‧d^−1^‧Pa^−1^; Water vapor permeability: 5.29 × 10^−6^ g‧m‧m^−2^‧d^−1^‧Pa^−1^). The “Jasmine” fresh table grapes were bought from an agricultural cooperative in Hohhot, China.

### 2.2 Preparation of Plant EOs Microcapsules (PEMC)

**FIGURE 1:**
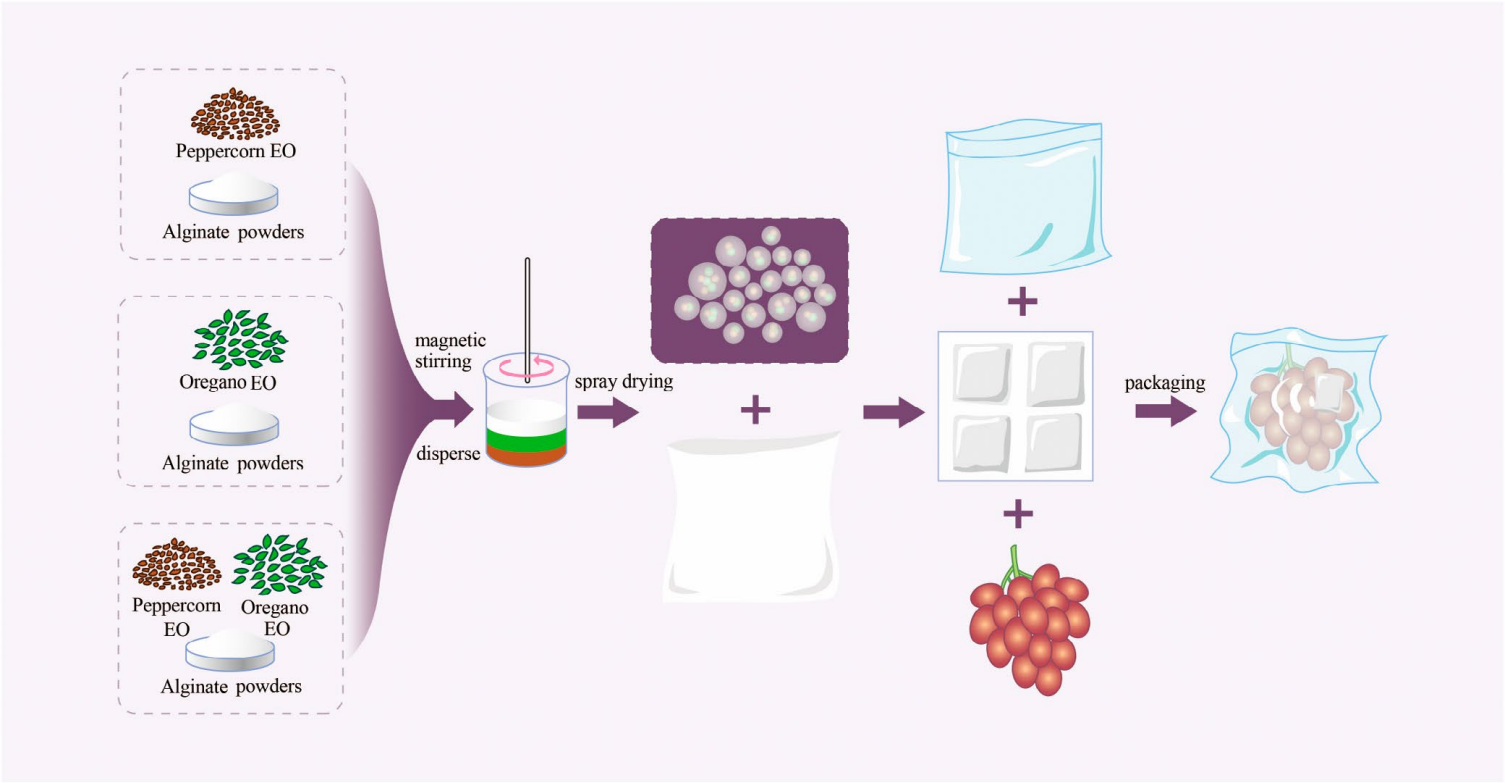
Preparation of plant EO micro capsules (PEMC) and the preservation of fresh table grapes.

The plant essential oil microcapsules (PEMC) were prepared using spray drying technology (Figure 1). A few modifications were made to the preparation process of the PEMC described by Wanli et al. (2022). To make a microencapsulated solution, the encapsulating material solution with sodium alginate at a mass fraction of 1.5% and the core material solution with essential oil at a volume fraction of 0.25% were prepared, respectively. The essential oils used in this study were PEO, OEO, and a 3:2 combination of PEO and OEO. The resultant mixture was then homogenized at 10,000 rpm for 20 min to form a uniform emulsion. For the spray drying process, a peristaltic pump was used to feed the emulsion at the pump flow rate of 15 mL·min^−1^, with a needle opening interval of 5 s. The inlet temperature of the spray dryer was maintained at 180°C, and the fan frequency was set to 50 Hz. The experimental conditions were controlled at a humidity level of (65 ± 2)% and a temperature of (23 ± 2)°C. Each microcapsule variant was characterized immediately following the spray drying process and was stored at 4°C in a sealed polyethylene bag within a plastic container until further analysis.

### 2.3 Characterization of Microcapsules Containing PEO, OEO, and a Combination of PEO and OEO

#### 2.3.1 Fourier Transform Infrared (FTIR) Analysis

The microcapsule samples and liquid essential oils were characterized using a FTIR spectrometer (IRAffinity‐1, Japan) fitted with an attenuated reflectance (ATR) device. The microcapsule samples were placed on a diamond crystal, and spectra were recorded with a resolution of 4 cm^−1^ over the range of 4000 cm^−1^ to 400 cm^−1^. A total of 64 scans were conducted, and fingerprint spectra were acquired at a resolution of 2 cm^−1^ within the range of 1800 to 600 cm^−1^.

#### 2.3.2 In Vitro Release Performance

To evaluate the impact of microencapsulation and MAP on the volatility characteristics of EOs, the release rate of EOs for different treatment groups was determined using a gravimetric method (Fenghua et al. 2022). The samples were divided into three main categories for subsequent experiments, with each group containing 5 g of the essential oils. The first unencapsulated essential oil group, included Peppercorn essential oil (PEO), oregano essential oil (OEO), and composite essential oil (CEO, a mixture of PEO and OEO at a mass ratio of 3:2). The second unpackaged microcapsule group, included PEO microcapsules, OEO microcapsules, and CEO microcapsules; the third microcapsule + PCL/PLLA film packaging group, included PEO microcapsules sealed with PCL/PLLA film, OEO microcapsules sealed with PCL/PLLA film, and CEO microcapsules sealed with PCL/PLLA film. All the above samples were maintained under consistent conditions (12°C ± 1°C) and weighed at 0, 2, 3, 5, 8, 9, 10, 14, and 18 days, respectively. The release rate (RR) was calculated using the following formula:

(2) $$\mathrm{RR}\%=\frac{\text{Initial weight of}\;\mathrm{EOs}-\text{testing weight of}\;\mathrm{EOs}}{\text{Initial weight of}\;\mathrm{EOs}}\times 100$$

#### 2.3.3 Scanning Electron Microscopy Analysis (SEM)

The morphology and structure of the microcapsules were observed using a scanning electron microscope (SEM, TM4000, Japan) in accordance with the methodology proposed by Tian et al. (2024). An appropriate amount of microcapsule powder was dipped with a cotton swab and then were gently placed on the surface of the conductive tape. The samples were coated with conductive adhesive on the copper plate and then stored in a vapor deposition chamber for gold plating. The microstructures of samples were observed using SEM with a magnification of 2000 times at an accelerating voltage of 15 kV.

### 2.4 Material Preparation and Processing Applications

**FIGURE 2:**
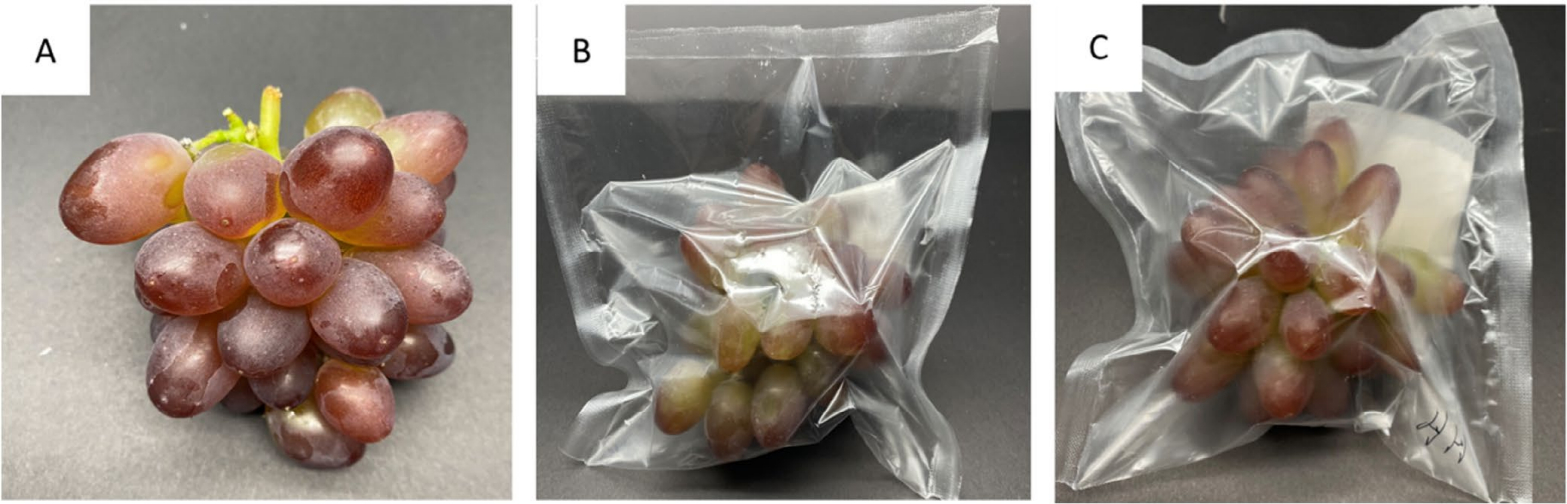
Different packaging treatment groups: (A) CK group, (B) PCL/PLLA group, (C) PCL/PLLA combined with CEOM group.

Grape clusters were transported in insulated containers in a car and pre‐cooled at 0°C–4°C for 4 h in a cold fresh cabinet. All mechanically damaged bunches were discarded, and only those exhibiting uniform size and color were selected. The selected bunches were immersed in a 5% hypochlorous acid solution for 3 min to eliminate surface microorganisms. Each grape cluster, weighing (150 ± 10) g, was then wrapped in PCL/PLLA film (dimensions: 16 × 17 cm). The sealing process was carried out using a multilayer heat sealer (DBF‐900, Zhejiang, China) under various packaging conditions. Microencapsulated essential oils were placed in nonwoven bags and co‐packaged with the grape bunches (Figure 2).

All table grape samples were maintained at a temperature of (12 ± 1)°C and a relative humidity of 85% for a duration of 28 days within a cold cabinet (KGES‐1200, Guangzhou, China), which was devoid of any other fruits or vegetables. Upon completion of the storage period, the grape samples underwent evaluation. Throughout the storage interval, a thorough analysis of their physical and chemical quality was performed.

The experimental groups are delineated as follows:

- PCL/PLLA group: packaged with PCL/PLLA films without the inclusion of microencapsulation.
- PCL/PLLA + PEOM group: packaged with PCL/PLLA films combined with 2 g peppercorn EO microcapsules in a non‐woven bag.
- PCL/PLLA + OEOM group: packaged with PCL/PLLA films combined with 2 g Oregano EO microcapsules in a non‐woven bag.
- PCL/PLLA + CEOM group: packaged with PCL/PLLA films combined with 2 g compound EO microcapsules in a non‐woven bag.
- CK group: Control Group (without packaging).

### 2.5 Headspace Gas Composition in Packaging

The concentrations of oxygen (O_2_) and carbon dioxide (CO_2_) within the packaging were monitored during storage using a headspace O_2_/CO_2_ analyzer (Model 3, MOCON, USA). Just before measurement, the packages were removed from storage and silicone septa were stuck to the film surfaces. Then, needles attached to the analyzer were inserted into the packages. The instrument was calibrated by reference to air before use. The gas composition of each package was measured, and the results are reported as expected percentages of air composition.

### 2.6 Evaluation of Table Grape Quality

#### 2.6.1 Sensory Assessment

The sensory evaluation was conducted in accordance with the methodology proposed by Giacosa et al. (2015). Briefly, a sensory evaluation panel consisting of 10 males and 10 females, all of whom have received training in sensory assessment, evaluated the different treatment groups of table grapes in terms of appearance, texture, aroma, and overall acceptability during storage in our sensory evaluation laboratory. The sensory evaluation was categorized into five levels: 0–2 points indicating “extremely unacceptable”; 2.1–4 points denoting “moderately unacceptable, loss of edibility”; 4.1–6 points representing “neutral”; 6.1–8 points referring to “moderately acceptable”; and 8.1–10 points signifying “acceptable.”

It is not a common practice for our institution to obtain permission for sensory panel research as an ethics policy regarding such subjects has not been established. Nevertheless, we can affirm that all necessary measures were taken to safeguard the rights and privacy of every participant throughout the research process. For instance, participants were not forced to take part; they were fully informed about the study's requirements and potential risks, their written or oral consent was obtained, their personal data was not disclosed without their awareness, and they had the right to withdraw from the study at any point.

#### 2.6.2 Determination of Weight Loss and Hardness

Weight loss (WL) refers to the percentage of the initial weight of fresh table grape bunches (Fenghua et al. 2022). After peeling, the hardness of the grapes was measured using a texture analyzer (TA‐XT Plus, Beijing, China). The test conditions were as follows: the testing speed was set at 1 mm/s, and the compressive deformation of the grape berries was 20%. Each berry was compressed twice, with a trigger force of 5 g. There was an intermediate pause of 5 s, and a P/100 compression head was used. Each group consisted of 10 replicates.

#### 2.6.3 Determination of Flavor Changes

The volatile compounds present in the table grape berries from different treatment groups, particularly sulfides and various terpenes, were analyzed using a portable electronic nose (PEN3.5, Germany AIRSENSE). For the analysis, samples consisting of 10 g grape berries and 2 g Eos microcapsules were placed into 30 mL sample vials and sealed with polyethylene sealing film. The samples were then stored at room temperature for 2 h to allow for flavor release. The test conditions included a sensor cleaning time of 120 s, a sample determination interval of 3 s, a sample preparation time of 5 s, a sample injection flow rate of 400 mL/min, and an automatic zeroing time of 5 s. The sensor was cleaned at the end of each test. Each treatment group was tested in six replicates. Finally, data were selected for subsequent analysis based on a smaller fluctuation amplitude, specifically from 114 to 120 s. The results of the flavor of grapes were expressed in the form of a Radar chart and the response values of the electronic noses of grapes in each treatment group.

### 2.7 Microbiological Assessment

#### 2.7.1 Microbiological Analysis

The microbial loads of the table grape were evaluated following the methodology proposed by Wenguang et al. (2018). Specifically, 5 g of the sample were mixed with 45 mL of physiological saline and homogenized at 4°C. A 1 mL aliquot of the liquid was then serially diluted and inoculated onto potato dextrose agar (PDA) plates. The plates were incubated at 37°C for 48 h, after which the total fungal count (TFC) was determined. The results were expressed as CFU·g^−1^.

#### 2.7.2 Determination of Microbial Diversity

The microbial diversity detection process involved several main steps, including sample preparation, DNA extraction and PCR amplification, purification and pooling of PCR products, library preparation and quality control, and high‐throughput sequencing using the NovaSeq platform. Initially, the surface and internal tissues of each grape sample were uniformly swabbed using sterile cotton swabs, with four replicates per treatment group to ensure consistent sampling. DNA was then extracted from the collected samples using the CTAB method, and the purity and concentration of the extracted DNA were assessed using agarose gel electrophoresis. The DNA was subsequently diluted to a concentration of 1 ng/μL using sterile water for downstream applications. PCR amplification targeted the ITS1 region with the primers ITS5‐1737F and ITS2‐2043R, and reactions were carried out using Phusion High‐Fidelity PCR Master Mix with GC Buffer (New England Biolabs, USA). Following amplification, the PCR products were verified through 2% agarose gel electrophoresis. Successfully amplified products were then purified using magnetic beads and pooled according to concentration.

Library preparation was carried out using the TruSeq DNA PCR‐Free Sample Preparation Kit (Illumina, USA). The prepared libraries were quantified using both Qubit fluorometric quantification and quantitative PCR (Q‐PCR) to ensure accuracy and quality. After passing quality control, the libraries were sequenced on the Illumina NovaSeq 6000 platform, generating 250 bp paired‐end reads.

### 2.8 Determination of Total Polyphenols and Flavonoids

The total phenolic and flavonoid contents were determined using a spectrophotometric method (SP‐756P, Shanghai, China). Seedless grape samples (5 g) were ground in an ice bath, and 10 mL of 2 mol/L HCl‐methyl alcohol solution was added. The mixture was then brought to a final volume of 20 mL and extracted for 20 min in the ice bath, avoiding exposure to light. Subsequently, the supernatant was measured at 280 nm for total phenolics (Zhang et al. 2024) and at 325 nm for flavonoids (Indiarto et al. 2024). The total phenolic content was calculated using a standard curve prepared with gallic acid (*y* = 0.0393*x* − 1.217, *R* ^2^ = 0.9992). The flavonoid content was determined using a standard curve prepared with rutin (*y* = 0.0142*x* − 0.0152, *R* ^2^ = 0.9992).

### 2.9 Data Analysis

All experimental units were measured in triplicate, and the resulting data is expressed as mean ± standard deviation (SD). A one‐way analysis of variance (ANOVA) was performed to assess the differences among the mean values. The least significant differences were determined using the Tukey B test (*p* < 0.05) in SPSS 20.0 (IBM, USA).

## 3 Results and Discussions

### 3.1 Characterization of EOs Microcapsules

**FIGURE 3:**
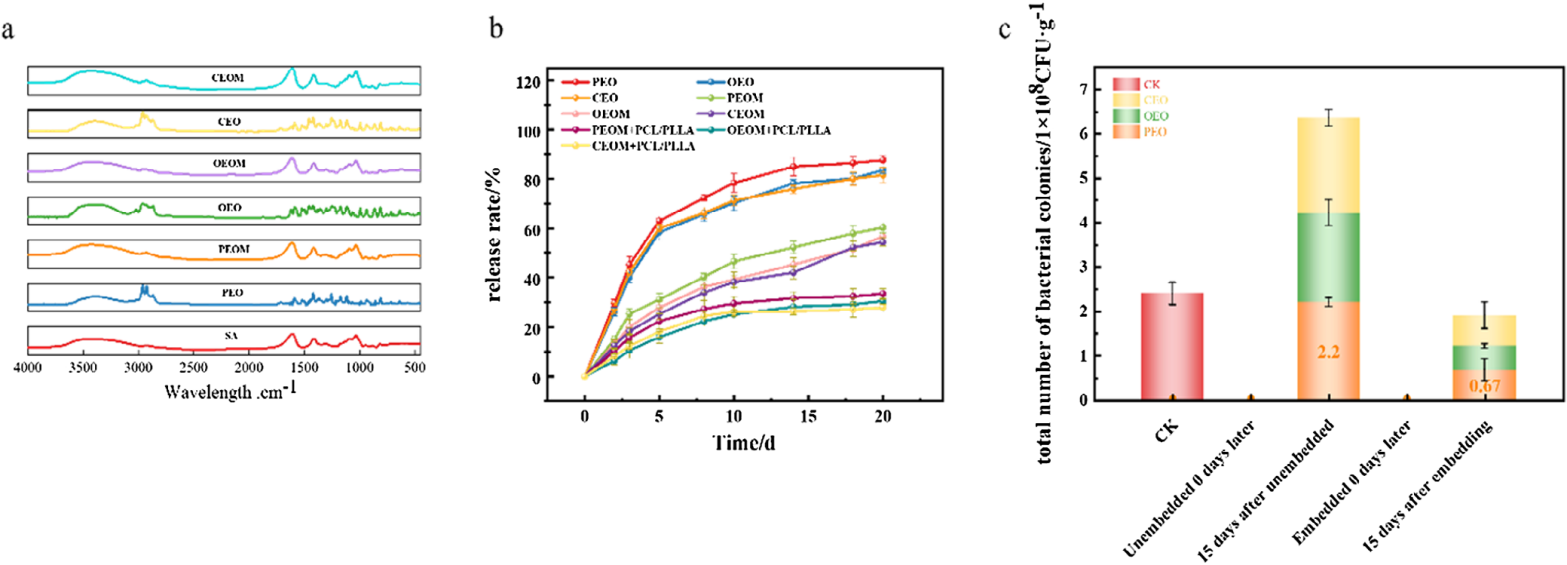
Characterization of pepper/oregano EO microcapsules: Infrared spectroscopy (a), release property analysis (b), antimicrobial property analysis (c).

Figure 3a presents the Fourier Transform Infrared Spectroscopy (FTIR) absorption spectra for sodium alginate (SA), peppercorn essential oil (PEO), oregano essential oil (OEO), composite essential oils (CEO), and their respective microcapsules (PEOM, OEOM, CEOM). Sodium alginate exhibits distinct absorption peaks at 1415 cm^−1^ and 1610 cm^−1^, corresponding to symmetric and asymmetric stretching vibrations of the ‐COO^−^ group, respectively. The peak at 1031 cm^−1^ is associated with C–O and C–C stretching vibrations of the pyranose ring. Additionally, the ‐OH stretching vibration is observed at 3406 cm^−1^, while the asymmetric C–H stretching in methylene groups peaks at 2926 cm^−1^ (Bokkhim et al. 2015).

PEO displays peaks at 2966 cm^−1^ (C–H stretching in alkanes) and 2856 cm^−1^ (symmetric C–H stretching in methylene groups). Other significant peaks include 1643 cm^−1^ (C=C stretching), 1452 cm^−1^ (in‐plane C–H bending), and 864 cm^−1^ (out‐of‐plane bending of olefin = CH_2_) (Na et al. 2018). OEO demonstrates peaks at 2960 cm^−1^ (flavonoid ‐OCH_2_ stretching) and 2735 cm^−1^ (C=O stretching in chain anhydrides), along with an additional peak at 1251 cm^−1^ (C–O–C ether stretching). Compared to pure SA, obviously, the broad ‐OH stretching peak of SA (3200–3600 cm^−1^) becomes wider or shifts to lower wavenumbers in the spectra of microcapsules (PEOM, OEOM, CEOM). This red shift and peak broadening occur because the ‐OH groups of SA form hydrogen bonds with polar groups in EOs (e.g., EO‐derived ‐OH or C=O), weakening the O‐H bond strength and altering its vibrational frequency. Thus, the spectral changes indicate the presence of hydrogen bonding interactions between EOs and SA, which facilitates stable encapsulation. In summary, FTIR spectroscopy confirms the successful microencapsulation of peppercorn EO, oregano EO, and their composite within SA‐based wall materials, and hydrogen bonding contributes to the interaction between EOs and the alginate matrix.

The FTIR spectra indicate that the microencapsulation profile of PEO closely resembles that of sodium alginate, confirming the successful encapsulation of PEO through the characteristic peaks at 2966 cm^−1^ and 2856 cm^−1^. Similar observations are noted for the OEO and composite EO groups (Zedong et al. 2021).

### 3.2 Release Rate Analysis of EOs Microcapsules

To investigate the effects of various treatments on the release rate of EOs, we quantified the release rate using a weighing method. Figure 3b illustrates the gradual volatilization of all groups of EOs over time. Notably, in open storage, the release rate exceeded 50% (w/w) by the 5th day. In contrast, the microencapsulation group exhibited a significantly slower release rate, suggesting the effectiveness of microencapsulation technology in reducing EO volatilization. Initially, there was a rapid release of EO within the first 5th day, which then slowed down. This deceleration can be attributed to the higher initial concentration within the microcapsules, facilitating rapid diffusion to the exterior at lower concentrations thereafter. This finding is consistent with the previous studies (Yu et al. 2024). EOs encapsulated with PCL/PLLA film resulted in the slowest release rate, as the enclosed environment maintained a dynamic equilibrium with the microcapsules, resulting in decreased EO release, as previously reported by Liu et al. (2023). This observation underscores the effective barrier properties of PCL/PLLA film against the three EOs.

### 3.3 Antimicrobial Efficacy of EOs Microcapsules

Figure 3c showed that on the 0th day, immediately after encapsulation, all groups of EOs exhibited notable antibacterial activity against *Escherichia coli* , with no colony formation observed in any group. After a 15‐day storage period at 12°C, the colony count for the non‐encapsulated EO group was (2.39 ± 0.45) × 10^8^ CFU·g^−1^, while the colony count for the encapsulated EO group was (6.30 ± 1.40) × 10^7^ CFU·g^−1^. Statistical analysis revealed a significant difference between the non‐encapsulated and encapsulated EO groups (*p* < 0.05). Additionally, we compared the colony counts among different EO types (CEO, OEO, PEO) within both non‐encapsulated and encapsulated groups; significant differences were also found (*p* < 0.05). These results indicated that the microencapsulated EOs retained substantial antibacterial efficacy even after 15 days. The use of sodium alginate for encapsulation was found to slow the volatilization rate of all three EOs, thereby providing prolonged antibacterial effects, which aligns with the findings of Peng et al. (2014).

### 3.4 Scanning Electron Microscopy (SEM) Analysis

**FIGURE 4:**
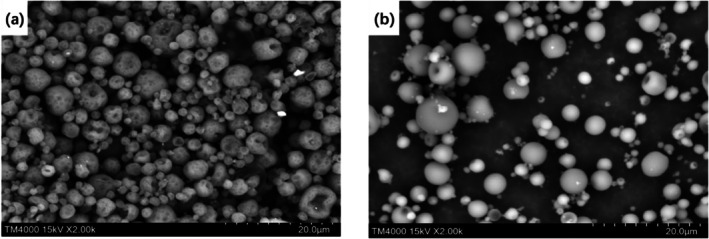
Scanning electron microscope images of the compound essential oil microcapsules after spray drying (a) and after washing off the surface oil (b).

As illustrated in Figure 4a, the spray‐dried EOs microcapsules are spherical in shape and exhibit an intact morphology without any surface cracks. This indicates that the EOs are effectively protected within the microcapsules. Figure 4b presents an image of the microcapsules after rinsing, which was done to remove any attached EOs. No observable changes in morphology were noted, further confirming that the EOs used in the subsequent preservation experiments originated from within the microcapsules.

### 3.5 The Quality Parameters of Grapes

#### 3.5.1 Appearance Quality

**FIGURE 5:**
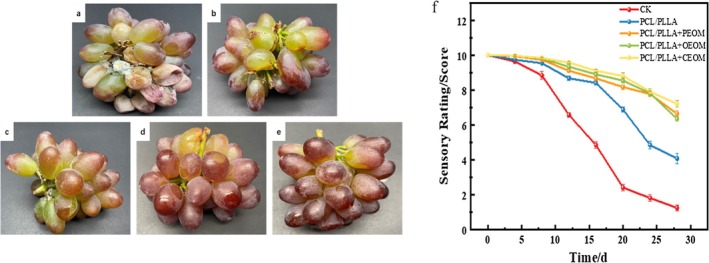
(a) C‐K; (b) PCL/PLLA; (c) PCL/PLLA+PEOM; (d) PCL/PLLA+OEOM; (e) PCL/PLLA+CEOM sensory pictures after 24 days of storage; (f) Sensory score.

Sensory quality plays a crucial role in consumer acceptance within the fruit and vegetable market. As shown in Figure 5f, the sensory scores of all treatment groups gradually declined throughout the grape storage period. Notably, the control (CK) group exhibited the most rapid decline, with a statistically significant difference from the other groups beginning on the 8th day (*p* < 0.05). The PCL/PLLA group also showed a sharp decrease in sensory score starting on the 20th day, which significantly differed from the PCL/PLLA + PEOM, PCL/PLLA + OEOM and PCL/PLLA + CEOM groups (*p* < 0.05), respectively. This trend is consistent with the appearance photos depicted in Figure 5a–e. Grapes in the CK group were stored without any packaging or treatment, leading to moisture loss from respiration and transpiration, resulting in wilting, browning, and discoloration that negatively affected sensory quality. In contrast, grapes in the PCL/PLLA group were stored in a low‐oxygen, high‐carbon dioxide atmosphere, which effectively inhibited the respiration of grapes and helped preserve their texture and color, resulting in higher sensory scores during the first 16 days of storage. However, the sensory score of grapes in the PCL/PLLA group declined more markedly than those of the essential oil treatment groups starting from the 20th day. This difference can be attributed to the sustained antibacterial effect of the essential oil microcapsules. The slow release of these compounds helped prevent grape rot and detachment from the rachis, thus maintaining a better visual appearance and sensory profile.

#### 3.5.2 Gas Composition in PMAP

**FIGURE 6:**
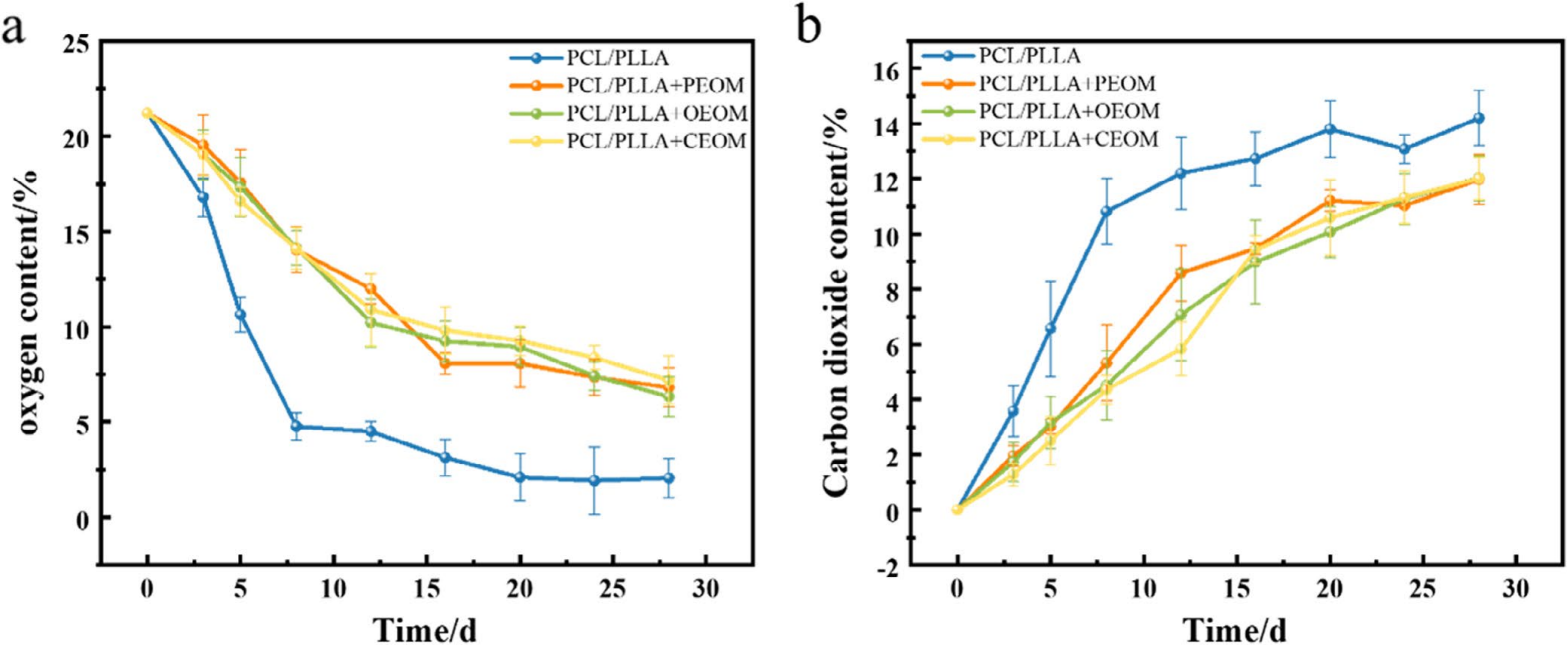
Concentration of O_2_ (a) and CO_2_ (b) inside the packaging during storage.

The gas composition within packaging is crucial for preserving the quality of fresh produce (Hyun and Lee 2017). As shown in Figure 6a, the oxygen concentration inside the packaging of all treatment groups gradually decreased as storage time increased, reaching a dynamic equilibrium by the 8th day. Notably, the oxygen concentration in the PCL/PLLA group dropped sharply beginning on the 4th day, showing a statistically significant difference compared to the essential oil treatment groups (*p* < 0.05). In contrast, no significant variation was observed among the essential oil combined with modified atmosphere packaging groups (*p* > 0.05), with oxygen levels stabilizing around 6.4%. This phenomenon may be attributed to the ability of essential oils to inhibit key respiratory enzymes, which slows the respiratory metabolism of the grapes. Consequently, the oxygen concentration remained higher in these treatments compared to the PCL/PLLA group, which lacked essential oils. Conversely, the carbon dioxide concentration inside the grape packaging showed a consistent upward trend throughout the storage period. A significant increase in CO_2_ concentration was observed in the PCL/PLLA group starting on the 8th day, which markedly differed from the groups treated with essential oils. However, no significant differences were detected among the essential oil combined packaging groups. This outcome further supports the hypothesis that essential oil treatments suppress respiratory activity, thus reducing CO_2_ accumulation (Yong et al. 2022).

#### 3.5.3 Weight Loss Analysis

**FIGURE 7:**
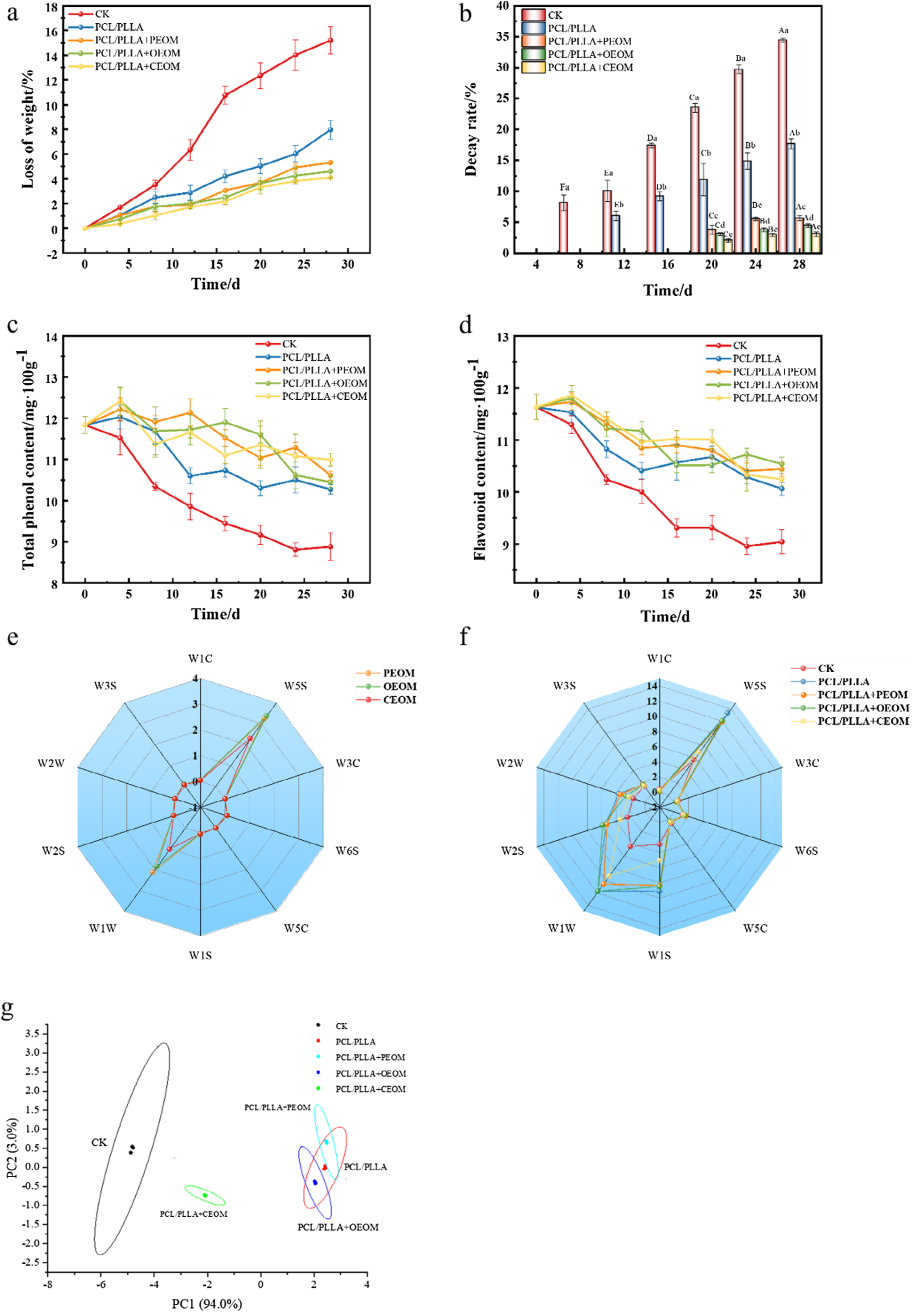
Analysis of physical and chemical indicators during storage of grapes: Analysis of weight loss rate (a), analysis of decay rate (b), analysis of total phenolics (c), analysis of flavonoid content (d), flavor test (e–g). Upper case letters indicate the significance of differences between storage days and lower‐case letters indicate the significance of differences between different treatment groups for the same storage time.

Fresh grapes contain over 80% moisture, making water their most abundant chemical component (Sun et al. 2024). This high moisture content is closely associated with sensory quality and physiological and biochemical characteristics. As illustrated in Figure 7a, the weight loss rate increased across all treatment groups throughout the storage period. By the 8th day, the CK group exhibited a weight loss rate of (3.52 ± 0.41)%, significantly higher than that of the other groups (*p* < 0.05). In the PCL/PLLA packaging group, the weight loss rate rose more gradually, likely due to the excellent water vapor barrier properties of PCL/PLLA films, which effectively reduce moisture loss. Beginning on the 12th day until the end of the storage period, the weight loss rate in the PCL/PLLA group increased significantly compared to the PCL/PLLA + PEOM, PCL/PLLA + OEOM and PCL/PLLA + CEOM groups (*p* < 0.05). By the 28th day, the weight loss rate in the PCL/PLLA group reached (7.90 ± 0.93)%, showing a statistically significant difference from the essential oil‐treated groups (*p* < 0.05). Among the treatment groups, the one containing CEOM demonstrated the lowest weight loss rate. This may be due to the antimicrobial properties of essential oils, which can inhibit microbial growth on grape surfaces, thereby reducing juice loss caused by decay. These findings suggest that the combination of EOs and PMAP can effectively reduce weight loss in grapes, which is consistent with the results reported by Teymoorian et al. (2024).

#### 3.5.4 Decay Rate Analysis

Mechanical damage and microbial contamination are major contributors to grape spoilage during postharvest storage, significantly affecting their market acceptance. As shown in Figure 7b, the decay rate in all treatment groups increased with prolonged storage time. By the 8th day, the decay rate in the control (CK) group reached (8.12 ± 1.85)%, which was significantly higher than that of the other treatment groups (*p* < 0.05). In the PCL/PLLA group, the rot rate climbed to (6.00 ± 1.30)% by the 12th day. In contrast, no signs of decay were observed in the groups that combined essential oils microcapsules with PMAP during the same period. By the 20th day, the rot rates for the PCL/PLLA + PEOM, PCL/PLLA + OEOM and PCL/PLLA + CEOM were (3.80% ± 0.78%), (3.10% ± 0.69%), and (2.10% ± 0.42%), respectively. These values were significantly lower than those of the untreated PCL/PLLA group (11.89% ± 2.29%) and the CK group (23.54% ± 4.15%) (*p* < 0.05). These findings demonstrate that PEOM, OEOM, and especially CEOM provide long‐term antimicrobial effects, significantly reducing grape rot during storage. The enhanced efficacy of the CEOM is likely due to the synergistic action of their components. These results align with previous research by Shi et al., which reported the successful use of oregano essential oil in preserving fresh apricots.

#### 3.5.5 Hardness Analysis

Fruit hardness is closely related to tissue structure and maturity, and it serves as a key indicator of postharvest quality in grapes. As shown in Table 1, the hardness of grapes in all treatment groups gradually declined over the storage period. Notably, the CK group exhibited the most significant reduction, with a 61.5% decrease in firmness from Day 0 to Day 28. This rapid decline is primarily attributed to the absence of any preservation treatment. Without packaging, respiration and transpiration were unregulated, accelerating the degradation of cell wall components such as cellulose and protopectin, thereby resulting in a marked loss of firmness. In comparison, the PCL/PLLA group showed a slower rate of firmness decline. On Day 12, grape firmness in this group remained at (264.7 ± 10.1) g, which was significantly higher than that of the CK group (*p* < 0.05). This preservation effect is likely due to the moderate gas permeability of the PCL/PLLA film, which helps maintain a relatively high CO_2_ environment inside the packaging. This, in turn, inhibits the activities of enzymes such as *β*‐galactosidase and pectinase, thus slowing the degradation of pectin in the cell wall. After 16 days of storage, the firmness of grapes in the PCL/PLLA + PEOM, PCL/PLLA + OEOM, and PCL/PLLA + CEOM groups remained in the range of 255–268 g, significantly higher than the (220.8 ± 8.5) g recorded in the PCL/PLLA group (*p* < 0.05). This enhanced firmness retention may be attributed to bioactive compounds such as pinene, sterols, and epoxy compounds present in pepper and oregano essential oils. These substances may act as inhibitors of *β*‐1,3‐glucanase and chitinase (Li et al. 2019), slowing their release during storage, reducing cell wall degradation, and thus preserving fruit firmness.

**TABLE 1:** Effect of treatments on the hardness of table grapes during storage.

Groups	Storage time/days							
	0	4	8	12	16	20	24	28
CK	304.4 ± 17.1g^Aa^	270.4 ± 19.2g^Ba^	250.2 ± 9.5g^Bab^	217.2 ± 10.4g^Cb^	152.9 ± 10.4g^Dc^	131.8 ± 5.9g^DEc^	120.4 ± 9.7g^Ec^	117.4 ± 10.2g^Ec^
PCL/PLLA	304.4 ± 17.1g^Aa^	285.8 ± 17.1g^ABa^	256.2 ± 11.3g^BCb^	264.7 ± 10.1g^Ca^	220.8 ± 8.5g^Db^	208.1 ± 10.3g^Db^	197.7 ± 11.2g^Db^	171.4 ± 14.1g^Eb^
PCL/PLLA + PEOM	304.4 ± 17.1g^Aa^	290.1 ± 16.4g^ABa^	275.0 ± 11.1g^BCab^	260.0 ± 11.6g^CDa^	255.0 ± 15.2g^CDEa^	239.8 ± 8.3g^DEa^	229.4 ± 12.3g^EFa^	211.5 ± 14.1g^Fa^
PCL/PLLA + OEOM	304.4 ± 17.1g^Aa^	285.2 ± 12.6g^ABa^	280.9 ± 10.2g^Ba^	274.7 ± 12.9g^Ba^	268.3 ± 9.7g^Ba^	239.2 ± 10.2g^Ca^	225.5 ± 12.3g^CDa^	213.8 ± 8.5g^Da^
PCL/PLLA + CEOM	304.4 ± 17.1g^Aa^	286.0 ± 14.8g^ABa^	275.4 ± 11.1g^Bab^	282.1 ± 10.9g^ABa^	268.1 ± 9.9g^Ba^	243.6 ± 10.3g^Ca^	234.8 ± 11.5g^Ca^	225.1 ± 11.5g^Ca^

*Note:* Upper case letters indicate the significance of differences between storage days and lower‐case letters indicate the significance of differences between different treatment groups for the same storage time.

#### 3.5.6 Flavor Changes Analysis

To investigate whether the flavor of grapes is affected by essential oil microcapsules combined with modified atmosphere packaging during storage, an electronic nose (PEN3.5, Germany, AIRSENSE) was employed for analysis (Zhao et al. 2024). Figure 7e presents the electronic nose radar profiles of the essential oil microcapsules, while Figure 7f displays the profiles of grapes from each treatment group after 12 days of storage. As shown in Figure 7f, the *G*/*G*_0_ values for two volatile‐sensitive sensors, W5S (sensitive to nitrogen oxides) and W1W (sensitive to sulfides and terpenes), were the highest across all treatment groups. The elevated response of the W1W sensor is likely due to its sensitivity to terpenes, which are abundant in both PEO and OEO. Correspondingly, Figure 7e indicates that the *G*/*G*_0_ values for the W1W sensor were 2.13 and 1.86 for PEOM and OEOM, respectively, both significantly higher than the 1.03 value for CEOM (*p* < 0.05). Figure 7g displays the results of principal component analysis (PCA) based on the electronic nose response values. The first principal component (PC1) accounted for 94.0% of the variance, and the second principal component (PC2) accounted for 3.0%, with a cumulative contribution rate of the two principal components reaching 97%, indicating that the PCA model adequately captured the differences in aroma profiles among the treatment groups. Variation in PC2 values suggested that the flavor characteristics of grapes were influenced by different preservation treatments. The overlapping positions of the PCL/PLLA, PCL/PLLA + OEOM, and PCL/PLLA + PEOM groups suggest similarities in their volatile profiles. Furthermore, Figure 7f shows that the *G*/*G*_0_ values for W1C (aromatic compounds, benzene), W3C (aromatic compounds, ammonia), and W5C (short‐chain alkanes) in the CK group were 0.36, 0.63, and 0.68, respectively, significantly higher than in the treated groups (*p* < 0.05). This may be attributed to the higher microbial load in the CK group, which can promote the production of volatile compounds such as alcohols, esters, and aromatics (Cecchini and Morassut 2010). Additionally, the *G*/*G*_0_ values of the W1W sensor for PEOM, OEOM and CEOM were 10.54, 11.77, and 9.27, respectively, while the PCL/PLLA group showed a value of 11.75. These results suggest that the use of essential oils did not increase the volatile intensity of the grapes, and in some cases, even led to a reduction. This reduction is likely due to the delayed ripening and senescence processes induced by essential oil treatment, resulting in decreased production of flavor‐related volatiles. In conclusion, CEOM produced fewer volatiles than the single PEOM and OEOM, indicating that the desired composite effect was achieved. Importantly, treatments with PEOM, OEOM and CEOM did not negatively affect the flavor of grapes, and may have reduced volatile content by delaying fruit maturation.

#### 3.5.7 Total Phenolic and Flavonoid Content Analysis

Total phenols and flavonoids contribute to pathogen resistance and exhibit strong antioxidant and free radical scavenging capacities (Robert et al. 2012). As shown in Figure 7c,d, the contents of total phenols and flavonoids in grapes gradually declined throughout the storage period. On the 8th day, the total phenol and flavonoid contents in the CK group decreased to (10.34 ± 0.57) mg·100 g^−1^ and (10.24 ± 0.48) mg·100 g^−1^, respectively. In contrast, the PCL/PLLA treatment group showed increases of 12.8% and 5.8% in total phenol and flavonoid contents compared to the CK group. By the 16th day, the PCL/PLLA + PEOM treatment group exhibited total phenol and flavonoid levels of (10.90 ± 0.79) mg·100 g^−1^ and (11.52 ± 0.96) mg·100 g^−1^, representing 17.08% and 21.90% increases over the CK group, respectively. At the end of the 24 days storage period, the total phenol and flavonoid contents in the PEOM and OEOM, and CEOM treatment groups were 4.7% and 3.37% higher than those in the PCL/PLLA group, and 21% and 15.1% higher than the CK group, respectively. The preservation of phenolic and flavonoid content is likely attributed to the synergistic effects of essential oil microcapsules combined with PMAP. This combination effectively reduces the respiration rate, inhibits the activity of degradative enzymes, and delays the senescence of grape fruits, thereby slowing the decline in total phenol and flavonoid levels. These findings are consistent with those reported by Du (2021), who demonstrated similar effects of grapefruit essential oil microcapsules on maintaining total phenol and flavonoid contents in fresh‐cut apples.

#### 3.5.8 Total Fungal Counts (TFC) Analysis

**FIGURE 8:**
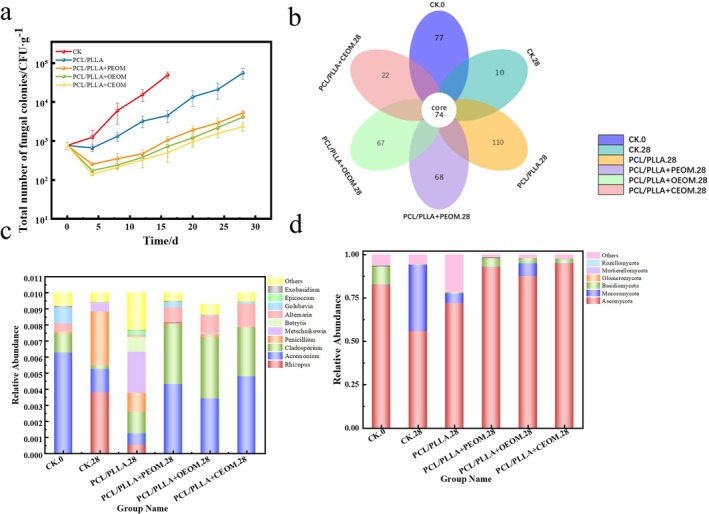
Analysis of microbiological indicators of grapes during storage, colony analysis (a), statistical analysis of fungal OUT (b), genus level (c), phylum level (d).

Figure 8a illustrated that the TFC in grapes across all treatment groups increased with prolonged storage time. In the CK group, significant decay was observed after 16 days of storage, with the total bacterial count reaching 4.9 × 10^4^ CFU·g^−1^. In contrast, the PCL/PLLA treatment group showed a reduction in microbial counts, decreasing from (7.60 ± 1.08) × 10^2^ CFU·g^−1^ to (6.63 ± 0.97) × 10^2^ CFU·g^−1^. This reduction may be due to the lower storage temperature rendering some microorganisms nonviable, along with the competition for oxygen within the packaging, which further inhibited microbial growth. Notably, the PCL/PLLA + PEOM, PCL/PLLA + OEOM, and PCL/PLLA + CEOM groups exhibited a more significant decrease in bacterial counts on the 4th day. As shown in Figure 7a, the TFC decreased to (2.52 ± 0.16) × 10^2^ CFU·g^−1^ for the PEOM group, (1.72 ± 0.14) × 10^2^ CFU·g^−1^ for the OEOM group, and (1.40 ± 0.11) × 10^2^ CFU·g^−1^ for the CEOM group on the 4th day, significantly lower than that in the CK group (6.62 ± 1.23) × 10^2^ CFU·g^−1^ (*p* < 0.05). This effect can be explained by the sustained release of encapsulated essential oils, which acted on the surface of grapes. On the other hand, the microorganisms that were not inactivated continued to proliferate at a suppressed rate. By Day 24 of storage, the TFC in the PEOM, OEOM, and CEOM groups was reduced by 90.1%, 93%, and 96%, respectively, compared to the PCL/PLLA group. These results strongly indicate that PEOM, OEOM and CEOM exert long‐term antibacterial effects. Notably, CEOM demonstrated superior antibacterial efficacy. These findings align with previous research by Du et al., which reported similar effects of pomelo essential oil microcapsules on the microbial load of fresh‐cut apples (Du 2021).

#### 3.5.9 Microbial Diversity Analysis

Table 2 showed that after 28 days of storage, the total number of fungal species in the CK group grapes was 87. In comparison, the species counts in the PCL/PLLA + PEOM, PCL/PLLA + OEOM, and PCL/PLLA + CEOM treatment groups were 165, 175, and 111, respectively, all lower than the 204 species observed in the PCL/PLLA group. To analyze the species composition further, sequencing data were clustered at a 97% similarity threshold, and the counts of fungal Operational Taxonomic Units (OTUs) were statistically analyzed across all treatment groups (Figure 8b). The results revealed that fresh grape samples contained 77 OTUs, which decreased to 10 after 28 days of storage. Grapes packaged with PCL/PLLA PMAP films exhibited 110 unique OTUs, which were reduced by approximately 38%–80% with the incorporation of essential oils. Figure 8c illustrated that at the phylum level, the dominant fungal groups included Ascomycota, *Mucoromycota*, *Basidiomycota*, *Glomeromycota*, *Mortierellomycota*, and *Rozellomycota*. Among these, *Ascomycota* was the most abundant across all groups, likely due to its prevalence in vineyard soil microbiota (Maglangit et al. 2018). After 28 days of storage, *Mucoromycota* increased to 38% in the CK group, which was associated with significant grape decay. This aligns with research indicating that *Mucoromycota* species are primary pathogens responsible for grape rot and mildew (Cui et al. 2024). As shown in Figure 8d, the CK group exhibited the highest post‐storage abundances of *Rhizopus* and *Penicillium*, accounting for approximately 38% and 34%, respectively. In contrast, the application of PCL/PLLA MAP film significantly reduced the abundances of *Penicillium* and *Rhizopus* to 12% and 4%, respectively (*p* < 0.05). Notably, the addition of PEOM reduced these abundances to 1% and 0%, respectively. OEOM achieved reductions to 2% and 0%, and the CEOM reduced both fungi to 0%. These results suggest that essential oil microcapsules, when combined with PMAP technology, effectively alter fungal community composition, suppress pathogenic fungi, and thereby enhance the postharvest quality and shelf life of *Jasmine* grapes.

**TABLE 2:** Determination of fungal α‐diversity index of grapes in each treatment group.

Group	OTUs	Shannon	Simpson	Chao1	ACE	Goods coverage
CK.0	126	2.4	0.7	152	158	0.9
CK.28	87	3.1	0.8	102	102	1
PCL/PLLA.28	204	3.3	0.8	247	253	0.9
PCL/PLLA + PEOM.28	165	2.9	0.8	187	197	0.9
PCL/PLLA + OEOM.28	175	3.0	0.8	201	215	0.9
PCL/PLLA + CEOM.28	111	3.6	0.9	122	123	1

The pronounced inhibition of *Penicillium* and *Rhizopus* by microcapsules incorporating both PEO and OEO can be attributed to the synergistic action of their key bioactive constituents (Jacqueline 2023; Di and Jia 2024). Hydrophobic molecules such as piperine and linalool from PEO, together with carvacrol and thymol from OEO, readily integrate into fungal cell membranes, compromising structural integrity and increasing permeability. This disruption leads to electrolyte leakage and the collapse of the proton motive force, impairing cellular energy conservation. Furthermore, phenolic compounds like carvacrol and thymol promote protein denaturation, while monoterpenes interfere with membrane lipid dynamics and disrupt respiratory enzyme function. An additional mechanism involves the induction of reactive oxygen species (ROS) accumulation, resulting in oxidative stress and inhibition of critical metabolic pathways (Yanlin et al. 2022). The multi‐targeted action exerted by the composite essential oils, simultaneously affecting membrane stability, energy metabolism, and protein function, explains the near‐complete suppression of spoilage fungi and underscores the synergistic efficacy observed in the CEOM treatment.

## 4 Conclusion

In this study, microcapsules of PEO and OEO were prepared using the spray‐drying method, and their physicochemical properties were thoroughly investigated. Fourier‐transform infrared spectroscopy provided confirmation of the successful encapsulation of the EOs. The microcapsules exhibited significant stability, and sustained release properties at room temperature. Meanwhile, the combination of EOs microcapsules and PMAP effectively inhibited the respiration of table grapes, reduced weight loss, maintained firmness, and suppressed the growth of spoilage microorganisms, thereby playing a crucial role in preserving post‐harvest quality and extending the shelf life of table grapes. This approach offers a practical solution for reducing post‐harvest losses and maintaining the commercial value of fresh table grapes.

## Author Contributions

**Xueyan Yun:** writing original draft, methodology, investigation, formal analysis. **Lu Zhang:** methodology, data curation. **Tungalag Dong:** resources, methodology, investigation. **Hongmei Zhang:** methodology, formal analysis. **Tao Sun:** visualization, validation. **Jian Hu:** software, methodology. **Peifang Cheng:** writing, review and editing, supervision, project administration, funding acquisition, conceptualization.

## Conflicts of Interest

The authors declare no conflicts of interest.

## Data Availability

Data will be made available on request.
